# Wild edible plants of Bhutan: Diversity, use patterns, and motivations for consumption

**DOI:** 10.1371/journal.pone.0355212

**Published:** 2026-08-28

**Authors:** Tenzin Jamtsho, Tobgay, Ugyen Takchu, Phurpa Wangchuk

**Affiliations:** 1 College of Science and Engineering, James Cook University, Cairns campus, McGregor Rd, Smithfield, Queensland, Australia; 2 Department of School Education, Yangchenphu High School, Ministry of Education (MoE), Thimphu, Bhutan; 3 Office of the Vice Chancellor, Royal University of Bhutan, Thimphu, Bhutan; 4 Department of Forest and Park Services, Jigme Khesar Strict Nature Reserve, Haa, Bhutan; 5 Australian Institute of Tropical Health and Medicine (AITHM), James Cook University, McGregor Rd, Smithfield, Cairns, Queensland, Australia; Government College Women University Sialkot: GC Women University Sialkot, PAKISTAN

## Abstract

Wild edible plants (WEPs) have long supported rural livelihoods and cultural practices in Bhutan, yet their use and associated knowledge are increasingly challenged by agricultural change, modernization, and limited documentation. This study documented the diversity, use patterns, and socio-ecological drivers of WEP use across Bhutanese communities in all 20 districts using purposive and snowball sampling of knowledgeable informants. Data were analyzed for species composition, life form, plant parts used, food categories, utilization trends, and ethnobotanical indices. A total of 114 WEP species belonging to 61 families were recorded. Urticaceae had the highest family importance value, herbs constituted the dominant life form (42%), and fruits were the most frequently used plant part (36.89%). Accessibility and availability were the main factors supporting continued use, whereas perceived ecological change, resource scarcity, land-use change, and limited knowledge constrained utilization. Fruits and vegetables dominated current use patterns, but responses also indicated declining knowledge transmission across generations. These findings highlight the continuing nutritional and cultural importance of WEPs in Bhutan and support the need for conservation, sustainable harvesting, and stronger integration of indigenous knowledge into education and policy.

## Introduction

Wild edible plants (WEPs), commonly referred to as bush foods, have long been integral to traditional food systems worldwide. The Food and Agriculture Organization (FAO) defines WEPs as plants that grow naturally in self-sustaining populations within natural or semi-natural ecosystems without direct human intervention [1]. Historically, they have played a crucial role in sustaining communities during periods of food scarcity, conflict, and natural disasters [2,3]. Research further shows that WEP use patterns vary markedly across cultural and geographic settings [4], highlighting their diverse functions and values. Even today, WEPs remain essential for approximately one billion people globally, especially in tribal and rural communities [5]. The edible parts of WEPs include inflorescences, shoots, buds, fruits, nuts, seeds, leaves, bark, stems, and roots [6,7], which are consumed either raw or prepared as spices and vegetables to suit local culinary preferences [8,9]. Wild edible fruits are particularly valued for their richness in micronutrients, vitamins, and antioxidants [10,11], while wild spices and vegetables contribute energy and essential nutrients that support dietary diversity and nutrition security [12].

Despite these benefits, global WEP use and knowledge are declining. Key drivers include urbanization, land-use change, agricultural expansion, loss of indigenous knowledge, sociocultural transformation, and climate change [5]. This trend has raised concerns amid rising food insecurity and biodiversity loss. As the world population is projected to exceed 9 billion by 2050, requiring a 50% increase in food production, experts suggest that the domestication and sustainable utilization of WEPs and other neglected species will be critical to future food systems [13].

Within this global discourse, Bhutan presents a distinctive ecological and cultural landscape for the study of WEPs. Situated in the eastern Himalayas and straddling the Indo-Malayan and Palaearctic biogeographic realms, Bhutan lies between China and India, two of the world’s most populous countries. The country is characterized by dramatic altitudinal variation, from 150 meters above sea level (masl) in the subtropical south to more than 7,500 masl in the northern alpine regions, resulting in highly diverse topography, ecosystems, and ecofloristic zones [13,14]. This diversity, together with substantial summer monsoon precipitation, places Bhutan within one of the world’s ten biodiversity hotspots [10].

Bhutan’s national development is guided by the philosophy of Gross National Happiness (GNH), which emphasizes environmental preservation alongside socioeconomic well-being. As a result, 71% of the country’s land area remains under forest cover, and 51% is protected through national parks, wildlife sanctuaries, nature reserves, and biological corridors [14]. However, the Flora of Bhutan has documented just over 5,603 species of flowering plants spanning 220 families and 1,415 genera, indicating substantial scope for further exploration. Approximately 94% of these species are native, and about 105 are endemic [7]. Many of these plants, including lichens, are traditionally used as medicinal plants and bush foods [15], contributing to both nutritional health and indigenous healing systems [16].

Despite this richness, wild edible plants in Bhutan have received limited research attention, as reflected in the small number of publications and policies devoted to them. As in other parts of the world, rapid urbanization and agricultural intensification have contributed to declines in both WEP consumption and the transmission of ethnobotanical knowledge [5,16,17]. In Bhutan, these shifts are compounded by environmental degradation, changing social values, and declining interest among younger generations [18,19]. Consequently, Bhutan risks losing the cultural and biological heritage associated with WEPs.

Against this backdrop, a nationally integrated assessment is needed to move beyond descriptive inventories and examine how WEP diversity, habitat association, use patterns, and socio-ecological drivers interact in Bhutan. This study therefore characterizes WEP diversity across communities, evaluates the cultural importance of taxa and families using ethnobotanical indices, assesses use patterns across food categories and plant parts, and identifies factors associated with continuing, increasing, moderate, or declining use.

Guided by ethnobotanical theory and global patterns of WEP use, we hypothesize that WEP importance is unevenly distributed across taxa, that forest-associated and herbaceous species make major contributions to use, and that land-use change and agricultural transitions are among the principal forces shaping contemporary WEP utilization. By addressing these questions, the study seeks to provide an evidence base for conserving biocultural diversity and supporting community-based resource management strategies in Bhutan.

## Methodology

### Study site and informant selection techniques

Located in the eastern Himalayas, Bhutan lies between 26.7263° and 28.2466° N and 88.7495° and 92.1236° E. It is bordered by India to the east, west, and south, and by China to the north. Administratively, the country is divided into 20 districts and 205 gewogs (blocks) [20]. Following a reconnaissance survey, data were collected across all 20 districts using purposive and snowball sampling to identify informants with recognized knowledge of WEPs. The final sample comprised 192 informants, including 20 key informants (16 male and 4 female) and 172 additional community informants (110 male and 62 female).

Key informants were selected on the basis of experience in identifying, collecting, and using WEPs, whereas additional informants were recruited to document more widely shared knowledge and to capture variation in knowledge transmission across age groups. Initial contacts were facilitated by district forest officials and local leaders, after which recruitment proceeded through community referral. The study employed non-interventional ethnobotanical interviews, home-based plant demonstrations, and guided field walks. Authorization and field clearance to conduct the study were granted by the Jime Khesar Strict Nature Reserve, Department of Forests and Park Services, Ministry of Agriculture and Forests, Royal Government of Bhutan (letter no. JKSNR/NCS-07/2022–2023/364, dated 16 March 2022). The Jigme Khesar Strict Nature Reserve coordinated and facilitated a field visit with relevant local authorities during data collection. All participants were informed about the purpose of the study, confidentiality procedures, the voluntary nature of participation, and their right to withdraw at any time without penalty, and prior informed consent was obtained from each participant before conducting the interviews.

Because the sampling design prioritized expertise rather than statistical balance, respondent numbers were uneven across districts. District-level patterns are therefore presented descriptively, whereas the main interpretation focuses on national-level patterns of diversity, utilization, and socio-ecological drivers.

### Questionnaire design

The questionnaire was developed in two sections. The first section addressed the socio-cultural attributes of the informants, including age, gender, and native origin. The second section focused on the traditional knowledge of informants regarding the uses of wild edible plants, covering aspects such as local and vernacular names, life forms, utilized parts, food categories, methods of usage, and modes of consumption. Expert review and feedback were sought on the questionnaires to ensure face and content validity prior to data collection.

### Data collection tools and procedures

Data were collected through semi-structured interviews, home-based plant demonstrations, and guided field walks. Interview schedules recorded local names, habitats, life forms, food categories, plant parts used, preparation methods, modes of consumption, and perceived changes in WEP use. Questions were prepared in English and, where necessary, administered in local languages to ensure accurate capture of local knowledge.

Home-based demonstrations were particularly useful for older informants with limited mobility, whereas other participants demonstrated collection and preparation practices in both household and field settings. Field surveys were conducted across all four seasons from March 2022 to December 2023, and specimens were collected in flowering or fruiting condition wherever possible. Photographs of live plants were taken, and specimens were processed using standard herbarium procedures recommended by Smith and Chinnappa [21]. Voucher specimens were deposited at the Jigme Khesar Strict Nature Reserve, Department of Forests and Park Services, Haa District, Bhutan. Taxonomic identification was verified against the Flora of Bhutan and other Himalayan references, and botanical names were cross-checked using World Flora Online, eFloras, and TROPICOS.

### Data analysis

Data were analyzed at the national level. Species diversity, life forms, habitats, food categories, plant parts used, and utilization trends were summarized using frequencies and percentages in figures and tables. Explanations for changes in WEP use were coded into environmental, economic, sociocultural, agricultural and land-use, and human-wildlife conflict domains; because informants could provide multiple explanations, Table 5 reports coded response frequencies rather than counts of unique informants. Chi-square tests were used to examine the association between WEP food categories and utilization trends, and chi-square goodness-of-fit tests were used to evaluate the distribution of coded responses within motivation domains.

Ethnobotanical indices, including Use Value (UV), Informant Consensus Factor (ICF), Relative Frequency Citation (RFC), Family Importance Value (FIV), and Fidelity Level (FL), were calculated following established methods [22–25].

The Family Importance Value (FIV) was determined by calculating the percentage of informants who referenced each family. It was computed as follows:


$$\begin{array}{c} \text{FIV}=\frac{\text{FC}(\text{family})}{\text{N}}\text{x100} \end{array}$$

where FC represents the count of informants mentioning the family, and N signifies the total number of informants involved in the study.

The Informant Consensus Factor was used to assess the consistency of knowledge regarding WEP use [26]. ICF is computed as follows:


$$\begin{array}{c} \text{ICF}=\frac{\text{nur}-\text{nt}}{\text{nur}-\text{1}} \end{array}$$

Where ‘nur’ represents the number of use reports for a given use category, and ‘nt’ indicates the number of taxa used for that category across all informants. ICF values are typically low (close to 0) when plant selection is random or when information exchange among informants is limited. Conversely, ICF values approach 1 when communities apply well-defined selection criteria or actively share knowledge [27].

To provide quantitative assessments of the relative importance of individual plant species to the informants, the Use Value (UV) was computed objectively [28]. This calculation was performed using the formula:


$$\begin{array}{c} \text{UV}=\frac{\sum \text{U}}{\text{n}} \end{array}$$

UV = ΣU/n, where UV denotes the use value of a species, U represents the number of use reports mentioned by the respondents, and n signifies the total number of respondents interviewed.

The Relative Frequency Citation (RFC) measured the frequency with which specific species were reported. It was calculated as follows:


$$\begin{array}{c} \text{RFC}=\frac{\text{FC}}{\text{N}} \end{array}$$

Where FC denotes the number of informants mentioning a particular WEP, and N represents the total number of informants surveyed. RFC values range from 0 to 1, with higher values indicating greater local importance of a species [25].

The Fidelity Level (FL) index was used to identify the extent to which a species was associated with a particular food category when the same species had multiple reported food uses. It is calculated as follows:


$$\begin{array}{c} \text{FL}=\frac{\text{Np}}{\text{N}}\text{ x 100} \end{array}$$

FL = (Np/N x 100). Here, Np represents the number of informants who cited a species for a particular food category, whereas N represents the total number of informants who cited that species for any food use. Higher FL values therefore indicate stronger agreement on the association of a species with a specific food category [24].

All ethnobotanical indices (RFC, FIV, UV, and FL) were calculated at the national level by aggregating responses across districts. This approach provided an overall assessment of WEP importance and use patterns in Bhutan. However, because the purposive and snowball sampling design produced uneven representation across districts, these indices were not further stratified by ecological region for inferential comparison. Such stratification would require balanced, systematically distributed sampling across regions to ensure statistical validity and comparability. The calculated indices are therefore interpreted as indicators of national-level patterns rather than region-specific differences.

Nevertheless, to explore whether citation values varied among ecological zones, the species citation data in Table 1 were matched to the ecological-zone categories reported in Table 2 and compared using a Kruskal-Wallis test. Because several zones were represented by very few species, the inferential comparison was restricted to the six zones with at least five recorded species (Ap, Bf, Brf, Cf, Stf, and Wbf).

**Table 1:** Wild edible plants recorded from different parts of Bhutan.

Family	Botanical name of the plants	Citation	RFC	Local names of the plants	Life form	Food Category	Parts used	Mode of consumption
Polygonaceae	*Aconogonon molle* (D.Don) H.Hara	1	0.01	Kochoma (tshangla)	H	salad	Sh	Eaten raw
Actinidiaceae	*Actinidia callosa* Lindl.	2	0.01	Phangulomsey (tshangla)	S	Fruit	Fr	Eaten raw
Acanthaceae	*Adhatoda vasica* Nees	4	0.02	Khatkali (khengkha)	H	Vegetable	F	Requires cooking
Primulaceae	*Ardisia macrocarpa* Wall.	2	0.01	Ressim (dzongkha)	S	Fruit	Fr	Eaten raw
Rosaceae	*Agrimonia pilosa* Ledeb	1	0.01	Nyobay puchen (mangdebikha)	H	salad	R	Eaten raw
Amaryllidaceae	*Allium wallichii* Kunth.	2	0.01	Lagop (dzongkha)	H	Vegetable, soup	Bu/F/Sh	Flavoring food, Extract juice/ raw
Betulaceae	*Alnus nepalensis* D.Don	1	0.01	Gamashing (dzongkha)	T	Dessert	Br	Eaten raw
Araceae	*Amorphophallus napalensis* (Wall.) Bogner & Mayo	1	0.01	Olkachu (dzongkha)	H	vegetables	Sh	Requires cooking
Thymelaeaceae	*Aquilaria malaccensis* Lam.	1	0.01	Agur (tshangla)	T	Fruit	Fr	Eaten raw
Asteraceae	*Artemisia dubia* Wall.	2	0.01	Khempa (dzongkha)	H	None	L/Ts/Sh	Local brew
Asteraceae	*Artemisia indica* Willd	1	0.01	Khempa (dzongkha)	H	None	L	Local brew
Asteraceae	*Artemisia vulgaris* L.	1	0.01	Khempa (dzongkha)	H	None	L/Ts/Sh	Local brew
Asparagaceae	*Asparagus racemosus* Willd.	1	0.01	Nalakhagchung (tshangla)	H	vegetables	Sh	Requires cooking
Poaceae	*Bambusa textilis* McClure	2	0.01	Boomba (khengkha)	S	vegetables	Sh	Requires cooking/ fermentation
Berberidaceae	*Berberis aristata* DC.	4	0.02	Kerpatsang (dzongkha)	S	Fruit	Fr	Eaten raw
Asteraceae	*Bidens pilosa* L.	1	0.01	Dhochan (khengkha)	H	Tea leaves/green tea	L	Prepare tea
Bombacaceae	*Bombax ceiba* L.	1	0.01	Pemageyser (dzongkha)	T	vegetables	L/F	Requires cooking
Araliaceae	*Brassaiopsis aculeata* Seem	1	0.01	Pangserpo (dzongkha)	T	Fruit	Ap	Eaten raw
Solanaceae	*Brugmansia suaveolens* (Humb. &Bonpl. exWilld.) Bercht. &J.Presl	1	0.01	Daw Gumju (lhotshampa)	S	soup	Ap	Plant parts can be smoked, eaten, drink as tea or taken as an enema
Arecaceae	*Calamus tenuis* Roxb	1	0.01	Menji (tshangla)	Li/Cl	Vegetable	Sh	Requires cooking
Brassicaceae	*Capsella bursa-pastoris* (L.) Medik.	1	0.01	Chamure Jhaar (lhotshampa)	H	vegetables	Wp	Requires cooking
Fabaceae	*Castanopsis hystrix* A. DC	1	0.01	Tsheshing (tshangla)	T	Salad, seeds	R	Fry and eat raw
Fabaceae	*Castanopsis indica* (Roxb. ex Lindl.) A.DC.	2	0.01	Tsha tsha sey (tshangla)	T	snack, fruits	R	Eaten raw / roasting
Rubiaceae	*Catunaregam longispina* Tirveng.	1	0.01	Nyertung sey	T	Fruit	Fr	Eaten raw
Apiaceae	*Centella asiatica* (L.) Urb.	1	0.01	Tuni Manakuni	H	leafy vegetable	Wp	Cooking
Rubiaceae	*Ceriscoides campanulata* Roxb	1	0.01	Desi	T	Fruit	Fr	Eaten raw
Rosaceae	*Chaenomeles speciosa* (Sweet) Nakai	1	0.01	Khoomangshing (tshangla)	T	Fruit	F	Extract juice
Anacardiaceae	*Choerospondias axillaris* (Roxb.) B.L.Burtt & A.W.Hill	1	0.01	Phrumchung sey (tshangla)	T	Fruit	Fr	Raw and make pickle
Lauraceae	*Cinnamomum zeylanicum* Blume	2	0.01	Chingchang (khengkha)	T	spices/tea	L/Br	Extract juice
Menispermaceae	*Cissampelos pareira* Linn.	1	0.01	Patha (dzongkha)	Li/Cl	Vegetable	Sh	Requires cooking
Rutaceae	*Citrus aurantiifolia* (Christm.) Swingle	1	0.01	Khomang (tshangla)	T	Fruit, spice	Fr/L	Fruit eaten raw, Fruit peel/leaves used as a spice in cooking
Rutaceae	*Citrus limon* (L.) Burm. f.	1	0.01	Kapur (tshangla)	T	Fruit	Fr	Eaten raw
Araceae	*Colocasia esculenta* (L.) Schott	3	0.02	Bozong (tshangla)	H	vegetables, snacks, and soup	Rh	Requires cooking
Cornaceae	*Cornus capitata* Wall.	3	0.02	Maminpa sey (tshangla)	T	Fruit	Fr	Eaten raw
Orchidaceae	*Cymbidium erythraeum* Lindl.	1	0.01	Karjan meto (tshangla)	H	Vegetable	F/Ts	Requires cooking
Orchidaceae	*Cymbidium floribundum* Lindl.	1	0.01	Olatshe (dzongkha)	H	Vegetable	F	Requires cooking
Fabaceae	*Dalbergia pinnata* Prain	1	0.01	Ollasema (dzongkha)	S	Fruit	Fr	Eaten raw
Thymelaeaceae	*Daphne bholua* D.Don	1	0.01	Shugu shing (tshangla)	S	Fruit	Fr	Eaten raw
Poaceae	*Dendrocalamus hamiltonii*	2	0.01	Pakshing (dzongkha)	S	Vegetable and salad	Sh	Requires cooking
Dioscoreaceae	*Dioscorea bulbifera* L	1	0.01	Tshemakewa (dzongkha)	Li/Cl	vegetables	Fr	Requires cooking
Dioscoreaceae	*Dioscorea hamiltonii* Hook.f.	3	0.02	Borang joktang (tshangla)	Li/Cl	Vegetables	Tu	Requires cooking
Ebenaceae	*Diospyros lotus* L	3	0.02	Gundum (dzongkha)	T	Fruit	Fr	Brewing alcoholic Fruit eaten raw
Athyriaceae	*Diplazium maximum* (D.Don) C.Chr.	3	0.02	Nakey (dzongkha)	H	Vegetable	Ap	Requires cooking
Sapotaceae	*Diploknema butyracea* (Roxb.) H.J.Lam	2	0.01	Fin sey (tshangla)	T	Fruit	Fr/Se	Raw and oil after extraction
Rosaceae	*Docynia indica* (Colebr. ex Wall.) Decne.	3	0.02	Thungkakpa sey (tshangla)	T	Fruit, Dessert	Fr	Raw/roast in fire/ sliced and dry/extract juice
Caryophyllaceae	*Drymaria cordata* (Linn.) Willd	1	0.01	Abhiijaalo (lhotshampa)	H	leaves	Wp	Heat and smell
Elaeagnaceae	*Elaeagnus latifolia* L	4	0.02	Dangmaling sey (tshangla)	S	Fruit	Fr	Eaten raw or after roasting
Urticaceae	*Elatostema lineolatum* Wight	9	0.05	Damburoo (tshangla)	H	Vegetable	Ap	Requires cooking
Moraceae	*Ficus auriculata* Lour	1	0.01	Chongma sey (tshangla)	T	Fruit	Fr	Fruit
Moraceae	*Ficus roxburghii* Miq	1	0.01	Chongma sey (tshangla)	T	Fruit	Fr	Eaten raw
Moraceae	*Ficus semicordata* Buch.- Ham. ex Sm.	2	0.01	Barchongma sey (tshangla)	T	Fruit	Fr	Eaten raw
Rosaceae	*Fragaria nubicola* (Lindl. ex Hook.f.) Lacaita	2	0.01	Brirtasazin (dzongkha)	H	Fruit	Fr	Eaten raw
Ericaceae	*Gaultheria fragrantissima* Wall.	1	0.01	Shakchungma sey (tshangla)	H	Fruit	Sh	Eaten raw
Gentianaceae	*Gentiana robusta* King ex Hook.f	1	0.01	Kyilchedkarpo (dzongkha)	H	Salad	F	Eaten raw
Urticaceae	*Girardinia diversifolia* Friis	2	0.01	Tsakusha (tshangla)	H	Soup or leafy vegetables	Ap	Requires cooking, extract juice, drying, and use for tea
Elaeagnaceae	*Hippophae salicifolia* D. Don	1	0.01	Starbu (dzongkha)	S	Fruit	Fr	Eaten raw, extract juice
Elaeagnaceae	*Hippophae tibetana* Schltdl	1	0.01	Starbu (dzongkha)	T	Jam, juice, Fruit	Fr	Extract juice
Lardizabalaceae	*Holboellia latifolia* Wall.	2	0.01	Throkchang sey (tshangla)	Li/Cl	Fruit	Fr	Eaten raw
Saururaceae	*Houttuynia cordata* Thunb	3	0.02	Krat (kurtoep)	H	salad, vegetables	Wp	Eaten raw or cooked
Schisandraceae	*Illicium verum* Hook.f.	2	0.01	Sekpa seng (tshangla)	S	Dessert, spices for cooking	Fr	Raw or cooked
Juglandaceae	*Juglans regia* L	2	0.01	Khesey (tshangla)	T	Fruit, snack	Fr	Seeds are eaten raw or after Roasting, and flowers are used as tea leaves
Acanthaceae	*Justicia adhatoda* L.	4	0.02	Khatsarim (tshangla)	H	Vegetable	F	Requires cooking
Liliaceae	*Lilium nanum* Klotzsch	1	0.01	Abikha (dzongkha)	H	Vegetables	Bu/F/Sh	Cooking
Lauraceae	*Litsea cubeba* (Lour.) Pers	2	0.01	Nenshing (tshangla)	T	Fruit, salad	Fr	Eaten raw as a salad and powdered as a spice
Lauraceae	*Litsea glutinosa* (Lour.) C. B. Rob	1	0.01	Kherim sey (tshangla)	T	Extract oil	Fr	Fruit
Lauraceae	*Machilus edulis* King ex Hook. f	1	0.01	Guli (tshangla)	T	Dessert	Fr	Fruit
Asparagaceae	*Maianthemum purpureum* (Wall.) LaFrankie	1	0.01	Ganchu gyem (dzongkha)	H	Vegetable	L	Requires cooking
Lamiaceae	*Mentha × officinalis* Hull	1	0.01	Nombarang (tshangla)	H	Mint Tea	L	Eaten raw
Moringaceae	*Moringa oleifera* Lam.	1	0.01	Bjinga (dzongkha)	T	Vegetable	Se	Extract juice, cooking,
Rutaceae	*Murraya koenigii* (L.) Spreng	1	0.01	Kari Patta (lhotshampa)	S	Fruit, spice	L/Fr	Raw and condiments in curry
Musaceae	*Musa × paradisiaca* L.	2	0.01	Laishing (tshangla)	H	Vegetables and fruits	Fr/F	Fruit ripened/ flowers cooked
Myricaceae	*Myrica esculenta* Buch.-Ham. ex D.Don	2	0.01	Tsutsu sey (tshangla)	T	Fruit	Fr	Eaten raw
Brassicaceae	*Nasturtium officinale* R.Br.	1	0.01	Jamakhatse (dzongkha)	H	vegetables and salad	Ap	Cooking and eaten raw
Plantaginaceae	*Neopicrorhiza scrophulariiflora* (Pennell) Hong	1	0.01	Putishing (dzongkha)	H	Soup	Tu	Boil and drink
Davalliaceae	*Nephrolepis cordifolia* (L.) C.Presl	2	0.01	Pangkay (dzongkha)	Fe	Fruit	Fr/Ber	Eaten raw
Apiaceae	*Oenanthe javanica* (Bl.) DC	1	0.01	Zhimtsi (dzongkha)	H	vegetables	Ap	Requires cooking
Boraginaceae	*Onosma hookeri* C.B. Clarke	1	0.01	Tsendaling	H	snack	R	Eaten raw
Bignoniaceae	*Oroxylum indicum* (L.) Kurz	3	0.02	Namkaling (tshangla)	T	Vegetable	F/Bu	Requires cooking
Rubiaceae	*Paederia foetida* L	1	0.01	Kheeru (tshangla)	Li/Cl	vegetables	Ap	Requires cooking or can be eaten raw as a side dish
Araliaceae	*Panax pseudoginseng* Wall	1	0.01	Bhreenggeeradza (dzongkha)	H	Soup	Tu	Boil and drink
Lauraceae	*Parasassafras confertiflora* (Meisn.) D. G. Long	2	0.01	Sey shing (tshangla)	T	oil	R	Extract oil
Lauraceae	*Persea bootanica* (Meisn.) Kosterm	1	0.01	Guli (tshangla)	T	Fruit	Fr	Eaten raw
Acanthaceae	*Phlogacanthus thyrsiformis* (Roxb. ex Hardw.) Mabb.	1	0.01	Plumsengma (khengkha)	S	Vegetable	F	Requires cooking
Phyllanthaceae	*Phyllanthus emblica* L.	4	0.02	Chhorgen sey (tshangla)	S	Fruit	Fr	Eaten raw
Solanaceae	*Physalis peruviana* L	1	0.01	Pokpokma sey (tshangla)	H	Fruit	Fr	Eaten raw
Phytolaccaceae	*Phytolacca acinosa* Roxb.	1	0.01	Kashakani (dzongkha)	H	Vegetable	Ap	Requires cooking
Scrophulariaceae	*Picrorhiza scrophulariiflora* Pennell	1	0.01	Putishing (dzongkha)	H	Medicine	Rh	Soaking
Piperaceae	*Piper pedicellatum* C. DC	2	0.01	Pepeling (dzongkha)	Li/Cl	Salad and desserts	L	Eaten raw
Arecaceae	*Plectocomia himalayana* Griff	6	0.03	Patsa (dzongkha)	H	Soup, vegetables	Sh	Requires cooking
Lamiaceae	*Pogostemon amaranthoides* Benth.	1	0.01	Nam Tshodma (tshangla)	H	Salad	L	Flavoring food
Asparagaceae	*Polygonatum kansuense* Maxim. ex Batalin	1	0.01	Khiraunla (lhotshamkha)	H	Shoot	Sh	Requires cooking
Myrtaceae	*Psidium guajava* L.	4	0.02	Bepsey (tshangla)	T	Fruit	Fr	Eaten raw
Pteridaceae	*Pteris biaurita* L.	2	0.01	Nakay (dzongkha)	H	Vegetables	Sh	Requires cooking
Fagaceae	*Quercus griffithii* Hook.f. & Thomson ex Miq.	1	0.01	Benangshing (tshangla)	T	Fruit	R	Eaten raw
Anacardiaceae	*Rhus chinensis* Mill	2	0.01	Roptangshing (tshangla)	T	Fruit	Fr	Eaten raw
Grossulariaceae	*Ribes griffithii* Hook.f. & Thomson	1	0.01	-	S	Fruit	Fr	Eaten raw
Asparagaceae	*Rohdea nepalensis* (Raf.) N.Tanaka	3	0.02	Nakima (lhotshampa)	H	Vegetable	Sh	Requires cooking
Rosaceae	*Rubus ellipticus* Sm.	1	0.01	Sergong (tshangla)	Li/Cl	Fruit	Fr	Eaten raw
Actinidiaceae	*Saurauia napaulensis* DC.	1	0.01	Gogun (lhotshampa)	T	Fruit	Fruit	Raw
Cucurbitaceae	*Solena amplexicaulis* (Lam.) Gandhi	1	0.01	Ban Kankri (lhotshampa)	Li/Cl	Fruit	Fr	Eaten raw
Fabaceae	*Tamarindus indica* L.	3	0.02	Tetari (tshangla)	T	Fruit, vegetable	Fr	Eaten raw, fruits are eaten raw, and young shoots are cooked
Combretaceae	*Terminalia bellirica* (Gaertn.) Roxb.	1	0.01	Baru (dzongkha)	T	Fruit	Fr	Eaten raw
Combretaceae	*Terminalia chebula* Retz.	1	0.01	Aaru (dzongkha)	T	Fruit	Fr	Eaten raw
Menispermaceae	*Tinospora cordifolia* (Willd.) Miers ex Hook. f. & Thomson	3	0.02	Zhimplengma (khengkha)	Li/Cl	Appetizer	St	Extract juice
Asparagaceae	*Tupistra nutans* Wall.	2	0.01	Wangpem meto (dzongkha)	H	Vegetable and pickle	Ap/If	Requires cooking
Asparagaceae	*Tupistra wattii* (C.B.Clarke) Hook. f.	1	0.01	Nakeyma	H	Vegetables	If	Requires cooking
Urticaceae	*Urtica dioica* L.	1	0.01	Jazzu (tshangla)	H	Soup	L	Boil and drink
Urticaceae	*Urtica parviflora* Roxb.	1	0.01	Zopcha (dzongkha)	H	Vegetable	Ap	Requires cooking
Ericaceae	*Vaccinium retusum* (Griff.) Hook.f. ex C.B.Clarke	2	0.01	Shakshingma sey (tshangla)	S	Fruit	Fr	Fruit
Santalaceae	*Viscum album* L	1	0.01	Jashi (dzongkha)	Epi	Tea leaves/green tea	L	Green tea
Santalaceae	*Viscum nepalense* Spreng.	1	0.01	Nyashitheub (dzongkha)	Epi	Tea ingredients	Wp	Drink after preparing the tea
Rutaceae	*Zanthoxylum armatum* DC.	1	0.01	Ghee (tshangla)	T	Dessert	Fr	Flavoring food
Rutaceae	*Zanthoxylum caribaeum* Lam.	1	0.01	Ghee (tshangla)	T	Fruit	Fr	Roast/dry and grind to powder, and use as a spice for curry
Rhamnaceae	*Ziziphus jujube* Mill.	1	0.01	Khansaling sey (tshangla)	S	Fruit	Fr	Eaten raw

Life form: T tree, S shrubs, H Herbs, Li liana, Cli climber, Fe fern, Epi epiphyte part of plant used: W whole plant, Ap aerial plants, TS tender stem, Ber berry, B bulb, Br bark, Bu buds, Fr fruit, F flowers, La latex, L leaf, R root, Rh rhizome, St stem, Se seeds, Sh shoots, T tuber/corm

**Table 2:** Environmental and sociocultural attributes of the study area.

Botanical name of the plants	Specimen ID	Dzongkhag^1^	Latitude	Longitude	Altitude (m)	Ecology^2^	Ethnicity^3^	Indigenous language^4^	Religion^5^
*Aconogonon molle* (D.Don) H.Hara	TJ/001/MON/2024	Mon	27.278507	91.232336	1413	Cf	Sha	Ts	Bud
*Actinidia callosa* Lindl.	TJ/002/PUN/2024	Pun	27.659743	89.769167	1585	Brf	Pun	Dz	Bud
*Adhatoda vasica* Nees	TJ/003/SAJ/2024	S/J	26.810628	91.495621	221	Stf	Sha	Ts	Bud
*Ardisia macrocarpa* Wall.	TJ/004/TRO/2024	Tro	27.448961	90.431345	2500	Brf	Tro	Md	Bud
*Agrimonia pilosa* Ledeb	TJ/005/MON/2024	Mon	27.258597	91.158204	787	Cf	Sha	Ts	Bud
*Allium wallichii* Kunth.	TJ/006/MON/2024	Mon	27.495378	91.346231	2701	Brf	Sha	Ts	Bud
*Alnus nepalensis* D.Don	TJ/007/WAN/2024	Wan	27.500614	90.031599	1599	Brf	Sh	Dz	Bud
*Amorphophallus napalensis* (Wall.) Bogner & Mayo	TJ/008/LHU/2024	Lhu	27.655277	91.177466	1918	Cf	Kur	Ku	Bud
*Aquilaria malaccensis* Lam.	TJ/009/ZHE/2024	Zhe	26.851865	90.965879	188	Stf	Khe	Dz	Bud
*Artemisia dubia* Wall.	TJ/010/PEM/2024	Pem	27.045905	91.347951	1173	Brf	Sha	Ts	Bud
*Artemisia indica* Willd	TJ/011/HAA/2024	Haa	27.381245	89.292161	2785	Bf	Haa	Dz	Bud
*Artemisia vulgaris* L.	TJ/012/MON/2024	Mon	27.265035	91.159607	696	Cf	Sha	Ts	Bud
*Asparagus racemosus* Willd.	TJ/013/LHU/2024	Lhu	27.666379	91.180944	1623	Cf	Kur	Ku	Bud
*Bambusa textilis* McClure	TJ/014/PEM/2024	Pem	27.037535	91.407532	1279	Brf	Sha	Ts	Bud
*Berberis aristata* DC.	TJ/015/HAA/2024	Haa	27.370556	89.240656	3965	Juf	Haa	Dz	Bud
*Bidens pilosa* L.	TJ/016/TRO/2024	Tro	27.413769	90.505407	1674	Brf	Tro	Md	Bud
*Bombax ceiba* L.	TJ/017/WAN/2024	Wan	27.270359	90.038029	646	Stf	Nga	Dz	Bud
*Brassaiopsis aculeata* Seem	TJ/018/TAY/2024	T/Y	27.586673	91.494686	1763	Cf	Yan	Yt	Bud
*Brugmansia suaveolens* (Humb. &Bonpl. exWilld.) Bercht. &J.Presl	TJ/019/TRA/2024	T/G	27.137656	91.570918	2180	Brf	Sha	Ts	Bud
*Calamus tenuis Roxb*	TJ/020/TSI/2024	Tsi	27.182444	90.071117	516	Cf	Nga	Dz	Bud
*Capsella bursa-pastoris* (L.) Medik.	TJ/021/TRO/2024	Tro	27.356376	90.562769	1049	Brf	Tro	Md	Bud
*Castanopsis hystrix* A. DC	TJ/022/WAN/2024	Wan	27.509539	90.087862	2096	Cbf	Sh	Dz	Bud
*Castanopsis indica* (Roxb. ex Lindl.) A.DC.	TJ/023/TRO/2024	Tro	27.342483	90.579904	1080	Brf	Tro	Md	Bud
*Catunaregam longispina* Tirveng.	TJ/024/SAJ/2024	S/J	27.004136	92.025878	1183	Wbf	Sha	Ts	Bud
*Centella asiatica* (L.) Urb.	TJ/025/MON/2024	Mon	27.273409	91.147091	744	Cf	Sha	Ts	Bud
*Ceriscoides campanulata* Roxb	TJ/026/CHU/2024	Chh	26.927645	89.375769	853	Wbf	Lho	Lh	Hind
*Chaenomeles speciosa* (Sweet) Nakai	TJ/027/BUM/2024	Bum	27.544447	90.724369	2896	Raf	Bum	Bu	Bud
*Choerospondias axillaris* (Roxb.) B.L.Burtt & A.W.Hill	TJ/028/MON/2024	Mon	27.286753	91.115465	1130	Cf	Sha	Ts	Bud
*Cinnamomum zeylanicum* Blume	TJ/029/SAR/2024	Sar	26.867909	90.229184	356	Stf	Lho	Lh	Hind
*Cissampelos pareira* Linn.	TJ/030/SAM/2024	Sam	26.935775	89.087291	638	Stf	Lho	Lh	Hind
*Citrus aurantiifolia* (Christm.) Swingle	TJ/031/TSI/2024	Tsi	27.030187	90.111676	1061	Brf	Lho	Lh	Hind
*Citrus limon* (L.) Burm. f.	TJ/032/SAR/2024	Sar	26.892269	90.482966	299	Stf	Lho	Lh	Hind
*Colocasia esculenta* (L.) Schott	TJ/033/ZHE/2024	Zhe	26.851688	90.948058	299	Stf	Khe	Dz	Bud
*Cornus capitata* Wall.	TJ/034/MON/2024	Mon	27.278927	91.205946	1055	Cf	Sha	Ts	Bud
*Cymbidium erythraeum* Lindl.	TJ/035/WAN/2024	Wan	27.550192	90.138305	2462	Cbf	Sh	Dz	Bud
*Cymbidium floribundum* Lindl.	TJ/036/HAA/2024	Haa	27.147467	89.176493	2559	Wbf	Haa	Dz	Bud
*Dalbergia pinnata* Prain	TJ/037/WAN/2024	Wan	27.490873	89.931644	1354	Cf	Sh	Dz	Bud
*Daphne bholua* D.Don	TJ/038/THM/2024	Thi	27.491368	89.746676	3122	Mcf	Nga	Dz	Bud
*Dendrocalamus hamiltonii*	TJ/039/LHU/2024	Lhu	27.679017	91.176659	1258	Cf	Kur	Ku	Bud
*Dioscorea bulbifera* L	TJ/042/LHU/2024	Lhu	27.663406	91.221954	1615	Cf	Kur	Ku	Bud
*Dioscorea hamiltonii* Hook.f.	TJ/041/SAJ/2024	S/J	27.007248	92.031642	1380	Wbf	Sha	Ts	Bud
*Diospyros lotus* L	TJ/043/LHU/2024	Lhu	27.691944	91.162966	1623	Cf	Kur	Ku	Bud
*Diplazium maximum* (D.Don) C.Chr.	TJ/044/DAG/2024	Dag	26.928343	89.990117	1015	Wbf	Lho	Lh	Hind
*Diploknema butyracea* (Roxb.) H.J.Lam	TJ/045/MON/2024	Mon	27.262305	91.168513	684	Cf	Sha	Ts	Bud
*Docynia indica* (Colebr. ex Wall.) Decne.	TJ/046/TRA/2024	T/G	27.206408	91.599759	2160	Brf	Sha	Ts	Bud
*Drymaria cordata* (Linn.) Willd	TJ/047/TSI/2024	Tsi	27.019188	90.113758	1274	Brf	Lho	Lh	Hind
*Elaeagnus latifolia* L	TJ/048/BUM/2024	Bum	27.583785	90.746851	2768	Bf	Bum	Bu	Bud
*Elatostema lineolatum* Wight	TJ/049/CHU/2024	Chh	27.077506	89.587462	2093	Wbf	Dru	Dz	Bud
*Ficus auriculata* Lour	TJ/050/LHU/2024	Lhu	27.609619	91.211157	1177	Cf	Kur	Ku	Bud
*Ficus roxburghii* Miq	TJ/051/TSI/2024	Tsi	26.958851	90.167223	1780	Brf	Lho	Lh	Hind
*Ficus semicordata* Buch.- Ham. ex Sm.	TJ/052/LHU/2024	Lhu	27602021	91.213892	1128	Cf	Kur	Ku	Bud
*Fragaria nubicola* (Lindl. ex Hook.f.) Lacaita	TJ/053/HAA/2024	Haa	27.372419	89.341765	3833	Ap	Ng	Dz	Bud
*Gaultheria fragrantissima* Wall.	TJ/054/MON/2024	Mon	27.290403	91.297085	2512	Brf	Sha	Ts	Bud
*Gentiana robusta* King ex Hook.f	TJ/055/HAA/2024	Haa	27.379195	89.201073	4206	Ap	Haa	Dz	Bud
*Girardinia diversifolia* Friis	TJ/056/CHU/2024	Chh	26.886458	89.384771	550	Stf	Lho	Lh	Hind
*Hippophae salicifolia* D. Don	TJ/057/THM/2024	Thi	27.424826	89.649473	2433	Bf	Nga	Dz	Bud
*Hippophae tibetana* Schltdl	TJ/058/HAA/2024	Haa	27.372932	89.185993	4323	Ap	Haa	Dz	Bud
*Holboellia latifolia* Wall.	TJ/059/GAS/2024	Gas	27.810499	89.722583	2415	Wbf	Dru	Dz	Bud
*Houttuynia cordata* Thunb	TJ/060/TRA/2024	T/G	27.120199	91.630841	1813	Brf	Sha	Ts	Bud
*Illicium verum* Hook.f.	TJ/061/THM/2024	Thi	27.517766	89.762537	2305	Cbf	Nga	Dz	Bud
*Juglans regia* L	TJ/062/CHU/2024	Chh	27.031074	89.380677	1992	Wbf	Dru	Dz	Bud
*Justicia adhatoda* L.	TJ/063/TSI/2024	Tsi	27.013947	90.087154	1150	Brf	Lho	Lh	Hind
*Lilium nanum* Klotzsch	TJ/064/HAA/2024	Haa	27.471988	89.121464	4031	Ap	Haa	Dz	Bud
*Litsea cubeba* (Lour.) Pers	TJ/065/TRA/2024	T/G	27.130427	91.568152	2058	Brf	Sha	Ts	Bud
*Litsea glutinosa* (Lour.) C. B. Rob	TJ/066/TRA/2024	T/G	27.165072	91.611286	2208	Brf	Sha	Ts	Bud
*Machilus edulis* King ex Hook. f	TJ/067/MON/2024	Mon	27.309726	91.334797	1890	Brf	Sha	Ts	Bud
*Maianthemum purpureum* (Wall.) LaFrankie	TJ/068/PAR/2024	Par	27.556117	89.358127	4085	Ap	Nga	Dz	Bud
*Mentha × officinalis* Hull	TJ/069/SAJ/2024	S/J	26.858127	91.449539	898	Stf	Sha	Ts	Bud
*Moringa oleifera* Lam.	TJ/070/SAR/2024	Sar	26.867874	90.255116	327	Stf	Lho	Lh	Hind
*Murraya koenigii* (L.) Spreng	TJ/071/SAM/2024	Sam	26.926628	89.046943	332	Stf	Lho	Lh	Hind
*Musa × paradisiaca* L.	TJ/072/DAG/2024	Dag	26.953994	89.968442	724	Wbf	Lho	Lh	Hind
*Myrica esculenta* Buch.-Ham. ex D.Don	TJ/073/MON/2024	Mon	27.266398	91.381274	1297	Cf	Sha	Ts	Bud
*Nasturtium officinale* R.Br.	TJ/074/MON/2024	Mon	27.281513	91.343241	1971	Cf	Sha	Ts	Bud
*Neopicrorhiza scrophulariiflora* (Pennell) Hong	TJ/075/HAA/2024	Haa	27.337054	89.159593	4333	Ap	Haa	Dz	Bud
*Nephrolepis cordifolia* (L.) C.Presl	TJ/076/PEM/2024	Pem	27.046343	91.418655	1735	Brf	Sha	Ts	Bud
*Oenanthe javanica* (Bl.) DC	TJ/077/MON/2024	Mon	27.232669	91.196923	601	Cf	Sha	Ts	Bud
*Onosma hookeri* C.B. Clarke	TJ/078/THM/2024	Thi	27.487314	89.616852	2839	Bf	Nga	Dz	Bud
*Oroxylum indicum* (L.) Kurz	TJ/079/SAM/2024	Sam	26.899788	89.099993	444	Stf	Lho	Lh	Hind
*Paederia foetida* L	TJ/080/TRA/2024	T/G	27.271631	91.539727	1945	Brf	Sha	Ts	Bud
*Panax pseudoginseng* Wall	TJ/081/HAA/2024	Haa	27.445319	89.185567	3212	Bf	Haa	Dz	Bud
*Parasassafras confertiflora* (Meisn.) D. G. Long	TJ/082MON//2024	Mon	27.226073	91.274834	2025	Brf	Sha	Ts	Bud
*Persea bootanica* (Meisn.) Kosterm	TJ/083/TRO/2024	Tro	27.285209	90.585744	1365	Brf	Tro	Md	Bud
*Phlogacanthus thyrsiformis* (Roxb. ex Hardw.) Mabb.	TJ/084/SAR/2024	Sar	26.879517	90.294741	676	Stf	Lho	Lh	Hind
*Phyllanthus emblica* L.	TJ/085/WAN/2024	Wan	27.497433	89.942939	1354	Cf	Sh	Dz	Bud
*Physalis peruviana* L	TJ/086/TSI/2024	Tsi	26.987568	90.117005	1688	Brf	Lho	Lh	Hind
*Phytolacca acinosa* Roxb.	TJ/087/TRO/2024	Tro	27.478824	90.494469	1921	Brf	Tro	Md	Bud
*Picrorhiza scrophulariiflora* Pennell	TJ/088/BUM/2024	Bum	27.727161	90.709579	4169	Ap	Bum	Bu	Bud
*Piper pedicellatum* C. DC	TJ/089/MON/2024	Mon	27.079206	91.153022	1165	Brf	Sha	Ts	Bud
*Plectocomia himalayana* Griff	TJ/090/ZHE/2024	Zhe	26.960167	91.049901	1174	Wbf	Khe	Dz	Bud
*Pogostemon amaranthoides Benth.*	TJ/091/SAJ/2024	S/J	27.008528	92.028196	1239	Wbf	Sha	Ts	Bud
*Polygonatum kansuense* Maxim. ex Batalin	TJ/092/WAN/2024	Wan	27.453742	90.184951	2840	Bf	Sh	Dz	Bud
*Psidium guajava* L.	TJ/093/MON/2024	Mon	27.305947	91.222139	1138	Cf	Sha	Ch	Bud
*Pteris biaurita* L.	TJ/094/LHU/2024	Lhu	27.605281	91.216576	1281	Cf	Kur	Ku	Bud
*Quercus griffithii* Hook.f. & Thomson ex Miq.	TJ/095/TRA/2024	T/G	27.257481	91.500115	2221	Brf	Sha	Ts	Bud
*Rhus chinensis* Mill	TJ/096/PEM/2024	Pem	27.050824	91.388273	1142	Brf	Sha	Ts	Bud
*Ribes griffithii* Hook.f. & Thomson	TJ/097/LHU/2024	Lhu	27.668876	91.190364	1199	Cf	Kur	Ku	Bud
*Rohdea nepalensis* (Raf.) N.Tanaka	TJ/098/GAS/2024	Gas	27.962950	89.539912	4036	Ap	Dru	Dz	Bud
*Rubus ellipticus* Sm.	TJ/099/TAY/2024	T/Y	27.426367	91.563403	874	Cf	Yan	Yt	Bud
*Saurauia napaulensis* DC.	TJ/100/GAS/2024	Gas	27.752959	89.728472	1723	Wbf	Dru	Dz	Bud
*Solena amplexicaulis* (Lam.) Gandhi	TJ/101/MON/2024	Mon	27.122231	91.100855	1793	Brf	Sha	Ts	Bud
*Tamarindus indica* L.	TJ/102/SAR/2024	Sar	26.888428	90.245923	520	Stf	Lho	Lh	Hind
*Terminalia bellirica* (Gaertn.) Roxb.	TJ/103/WAN/2024	Wan	27.542407	90.101244	1944	Brf	Sh	Dz	Bud
*Terminalia chebula* Retz.	TJ/104/WAN/2024	Wan	27.358125	89.915822	1018	Brf	Sh	Dz	Bud
*Tinospora cordifolia* (Willd.) Miers ex Hook. f. & Thomson	TJ/105/SAR/2024	Sar	26.901853	90.346387	579	Stf	Lho	Lh	Hind
*Tupistra nutans* Wall.	TJ/106/WAN/2024	Wan	27.542697	90.158864	2674	Brf	Sh	Dz	Bud
*Tupistra wattii* (C.B.Clarke) Hook. f.	TJ/107/MON/2024	Mon	27.286413	91.317086	2275	Brf	Sha	Ts	Bud
*Urtica dioica* L.	TJ/108/SAM/2024	Sam	26.896768	89.108007	588	Stf	Lho	Lh	Hind
*Urtica parviflora* Roxb.	TJ/109/TAY/2024	T/Y	27.628389	91.496867	2031	Cf	Yan	Yt	Bud
*Vaccinium retusum* (Griff.) Hook.f. ex C.B.Clarke	TJ/110/MON/2024	Mon	27.369373	91.027279	3096	Fif	Sha	Ts	Bud
*Viscum album* L	TJ/111/TRO/2024	Tro	27.284333	90.611827	1484	Brf	Tro	Md	Bud
*Viscum nepalense* Spreng.	TJ/112/WAN/2024	Wan	27.499494	90.166243	3069	Bf	Sh	Dz	Bud
*Zanthoxylum armatum* DC.	TJ/113/MON2024	Mon	27.258544	91.175558	630	Cf	Sha	Ts	Bud
*Zanthoxylum caribaeum* Lam.	TJ/114/MON2024	Mon	27.106455	91.321036	1333	Cf	Sha	Ts	Bud
*Ziziphus jujube* Mill.	TJ/115/SAM/2024	Sam	26.908182	89.072928	325	Stf	Lho	Lh	Hind

^1^Bum: Bumthang, Chh: Chhukha, Dag: Dagana, Gas: Gasa, Haa:Haa, Lhu: Lhuntse, Mon: Mongar, Par: Paro, Pem: Pema Gatshel, Pun: Punakha, S/J: Samdrup Jongkhar, Sam: Samtse, Sar: Sarpang, T/G: Tashigang, T/Y: Tashiyangtse, Thi: Thimphu, Tro: Trongsa, Tsi: Tsiring, Wan: Wangdue, Zhe: Zhemgang

^2^Ap; Alpine, Bf; Bluepine forest, Brf; Broad leaf Forest, Cf; Chripine forest, Cbf; Cool Broad leaf Forest, Fif; Fir Forest, Juf; Junper forest, Mcf; Mixed Conifer Forest, Stf; Sub-tropical forest, Wbf; Warm broad leaf forest, Raf; riparian forest

^3^Bum; Bumthap, Dru; Drukpa, Haa; Haap, Khe; khengpa, Kur; Kurtoep, Lho; Lhostahmpa, Nga; Ngalop, Pun; Punap, Sh; Shaa, Tro; Trongsap, Sha; Sharchop, Yan; Yangtshep

^4^Bu; Bumthap, Dz; Dzongkha, Kh; Khenkha, Ku; Kurtoepkha, Lh; Lhotshamkha, Ts; Tshangla, Ch; Chalip, Md; Mangdip, Yt; Yangtshep

^5^Bud: Bhuddhist, Hind; Hindus

## Results and discussion

### Demography and social structure

Demographic information was collected across districts to characterize the respondent profile and contextualize ethnobotanical knowledge of WEPs, following Kumar et al. [29]. The respondents were distributed across all districts, providing broad national coverage of WEP knowledge and practices. However, the number of respondents varied considerably, ranging from 38 in Mongar to 1 each in Gasa and Paro. This disparity likely reflects differences in population size, settlement patterns, and the availability of recognized knowledge holders across regions.

**Fig 1:**
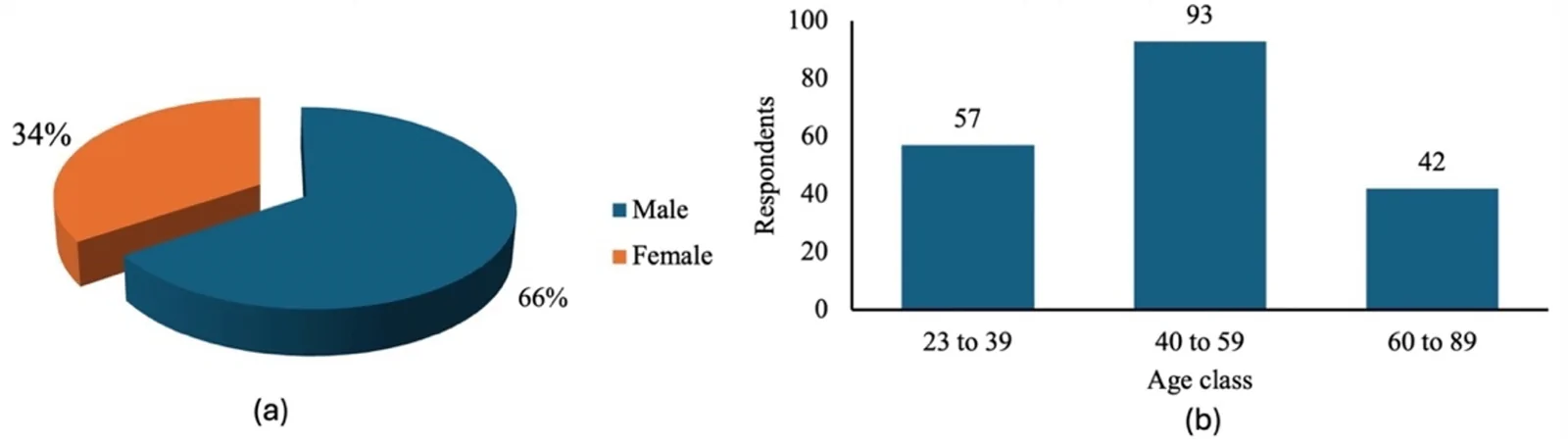
Respondents by gender (a) and age class (b).

Such variation in respondent distribution is an expected outcome of purposive ethnobotanical sampling, which prioritizes knowledgeable informants over statistically uniform representation. Nevertheless, this imbalance limits the robustness of direct district-level comparisons. District-level variation is therefore presented descriptively rather than interpreted as a statistical ranking of WEP richness or use intensity. Within this sampling context, gender may also shape ethnobotanical knowledge through differences in livelihood roles, access to resources, education, and social networks [30]. Although efforts were made to include both sexes, the respondent pool was male-skewed, with males accounting for 66% (n = 126) and females 34% (n = 66) (Fig 1a). This pattern suggests that the documented knowledge may partly reflect gendered differences in access to WEP collection and use and contrasts with studies reporting a stronger association between women and wild plant knowledge [24,27].

Age distribution further showed that respondents aged 40–59 years constituted the largest group, representing 48% (n = 93) of the total sample (Fig 1b). Individuals in this age group, predominantly male, demonstrated comparatively high levels of WEP knowledge. This pattern may reflect the Bhutanese context, in which men are more actively involved in field collection of wild plants, whereas women are often more engaged in household food preparation and nearby resource gathering. Similar observations have been reported elsewhere [31].

### WEP diversity and habitat distribution patterns

This ethnobotanical survey documented 114 WEP species across 61 families in 20 districts of Bhutan. Earlier studies from Bhutan focused mainly on wild edible fruits, non-wood forest products, or the ethnomedicinal properties of wild plants in specific regions. For example, Bajpai reported 120 WEPs from 63 families in southeastern Bhutan [32], whereas Yangdon recorded 52 wild edible plants from 47 genera and 35 families in eastern Bhutan [19]. The present study takes a broader approach by covering all major WEP food categories. Comprehensive information on family and botanical names, local names, life forms, plant parts used across food categories, modes of consumption, frequency citation (FC), and relative frequency citation (RFC) is presented in Table 1.

Among the recorded families, Lauraceae contained the highest number of species [6], followed by Rosaceae, Asparagaceae, and Asteraceae (5 each), while Fabaceae, Moraceae, and Urticaceae each contained 4 species (Table 3). Similar dominance of Moraceae, Rosaceae, Fabaceae, Lauraceae, Rutaceae, and Urticaceae has been reported in Uttarakhand, India [32], among the Gaddis, a tribal community of the western Himalaya [33], in Burji District, Ethiopia [22], in Chile [34], and in eastern Bhutan [19]. The remaining 56 families were represented by fewer than 3 species each (Tables 1 and 3). Details on frequency citation (FC), relative frequency citation (RFC), and family importance value (FIV) for each family are provided in Table 3.

**Table 3:** Family diversity and its importance as WEPs.

Family	No. taxa	%	Citation	RFC	FIV	Family	No. taxa	%	Citation	RFC	FIV
Anacardiaceae	3	2.54	4	0.021	2.08	Lauraceae	6	5.09	9	0.047	4.69
Asteraceae	5	4.24	6	0.031	3.13	Liliaceae	1	0.85	1	0.005	0.52
Acanthaceae	3	2.54	7	0.036	3.65	Melanthiaceae	1	0.85	1	0.005	0.52
Actinidiaceae	1	0.85	2	0.010	1.04	Menispermaceae	2	1.70	4	0.021	2.08
Amaryllidaceae	1	0.85	2	0.010	1.04	Moraceae	4	3.39	5	0.026	2.60
Apiaceae	2	1.70	2	0.010	1.04	Moringaceae	1	0.85	1	0.005	0.52
Araceae	1	0.85	4	0.021	2.08	Musaceae	1	0.85	2	0.010	1.04
Araliaceae	2	1.70	2	0.010	1.04	Myricaceae	1	0.85	2	0.010	1.04
Arecaceae	2	1.70	7	0.036	3.65	Myristicaceae	1	0.85	1	0.005	0.52
Asparagaceae	5	4.24	7	0.036	3.65	Myrtaceae	2	1.70	6	0.031	3.13
Athyriaceae	1	0.85	3	0.016	1.56	Orchidaceae	2	1.70	2	0.010	1.04
Berberidaceae	1	0.85	4	0.021	2.08	Phyllanthaceae	1	0.85	4	0.021	2.08
Betulaceae	1	0.85	1	0.005	0.52	Phytolaccaceae	1	0.85	1	0.005	0.52
Bignoniaceae	1	0.85	3	0.016	1.56	Piperaceae	2	1.70	2	0.010	1.04
Bombacaceae	1	0.85	1	0.005	0.52	Plantaginaceae	1	0.85	1	0.005	0.52
Boraginaceae	1	0.85	1	0.005	0.52	Poaceae	2	1.70	4	0.021	2.08
Brassicaceae	2	1.70	2	0.010	1.04	Polyganaceae	1	0.85	1	0.005	0.52
Caryophyllaceae	1	0.85	1	0.005	0.52	Primulaceae	1	0.85	2	0.010	1.04
Combretaceae	2	1.70	2	0.010	1.04	Pteridaceae	1	0.85	1	0.005	0.52
Cornaceae	1	0.85	3	0.016	1.56	Rhamnaceae	1	0.85	1	0.005	0.52
Cucurbitaceae	1	0.85	1	0.005	0.52	Rosaceae	5	4.24	8	0.042	4.17
Davalliaceae	1	0.85	2	0.010	1.04	Rubiaceae	3	2.54	3	0.016	1.56
Dioscoreaceae	3	2.54	5	0.026	2.60	Rutaceae	5	4.24	5	0.026	2.60
Ebenaceae	1	0.85	3	0.016	1.56	Santalaceae	2	1.70	2	0.010	1.04
Elaeagnaceae	3	2.54	6	0.031	3.12	Sapotaceae	1	0.85	2	0.010	1.04
Ericaceae	2	1.70	4	0.021	2.08	Saururaceae	1	0.85	3	0.016	1.56
Fabaceae	4	3.39	7	0.036	3.65	Schisandraceae	1	0.85	2	0.010	1.04
Gentianaceae	1	0.85	1	0.005	0.52	Scrophulariaceae	1	0.85	1	0.005	0.52
Grossulariaceae	1	0.85	1	0.005	0.52	Solanaceae	2	1.70	2	0.010	1.04
Juglandaceae	1	0.85	2	0.010	1.04	Thymelaeaceae	2	1.70	1	0.005	0.52
Lamiaceae	2	1.70	2	0.010	1.04	Urticaceae	4	3.39	12	0.063	6.25
Lardizabalaceae	1	0.85	2	0.010	1.04						

The application of plant family use value in ethnobotanical studies enables the assessment of the significance of different plant families, facilitating hypothesis testing, statistical validation, and comparative analysis [35]. Urticaceae, with an FIV of 6.250%, represented by four plant species, emerges as the most valuable family for wild edibles. Following Urticaceae, Lauraceae, Rosaceae, Acanthaceae, Arecaceae, Asparagaceae, and Elaeagnaceae also contribute substantially, reflecting their importance in local diets and traditional practices (Table 3). The suitability of habitats, favorable environmental conditions, and frequent interactions with local communities likely explain the dominance of these families [36]. Consequently, the traditional use of these plant species is widely recognized among the inhabitants.

While these indices provide valuable insights into the relative importance of plant families at the national scale, a supplementary ecology-stratified comparison of species citation values was undertaken by linking the species records in Table 1 to the ecological-zone categories in Table 2. Citation values did not differ significantly among the six sufficiently represented ecological zones (Kruskal-Wallis χ² = 5.12, df = 5, p = 0.402; n = 107 species), although warm broadleaved forest (Wbf) showed the highest median citation value. A sensitivity analysis using four broader ecological groups yielded the same conclusion (χ² = 1.73, df = 3, p = 0.630). Within the limits of the present sampling design, ecological variation in citation values is therefore interpreted descriptively rather than as evidence of statistically distinct among-zone differences.

**Fig 2:**
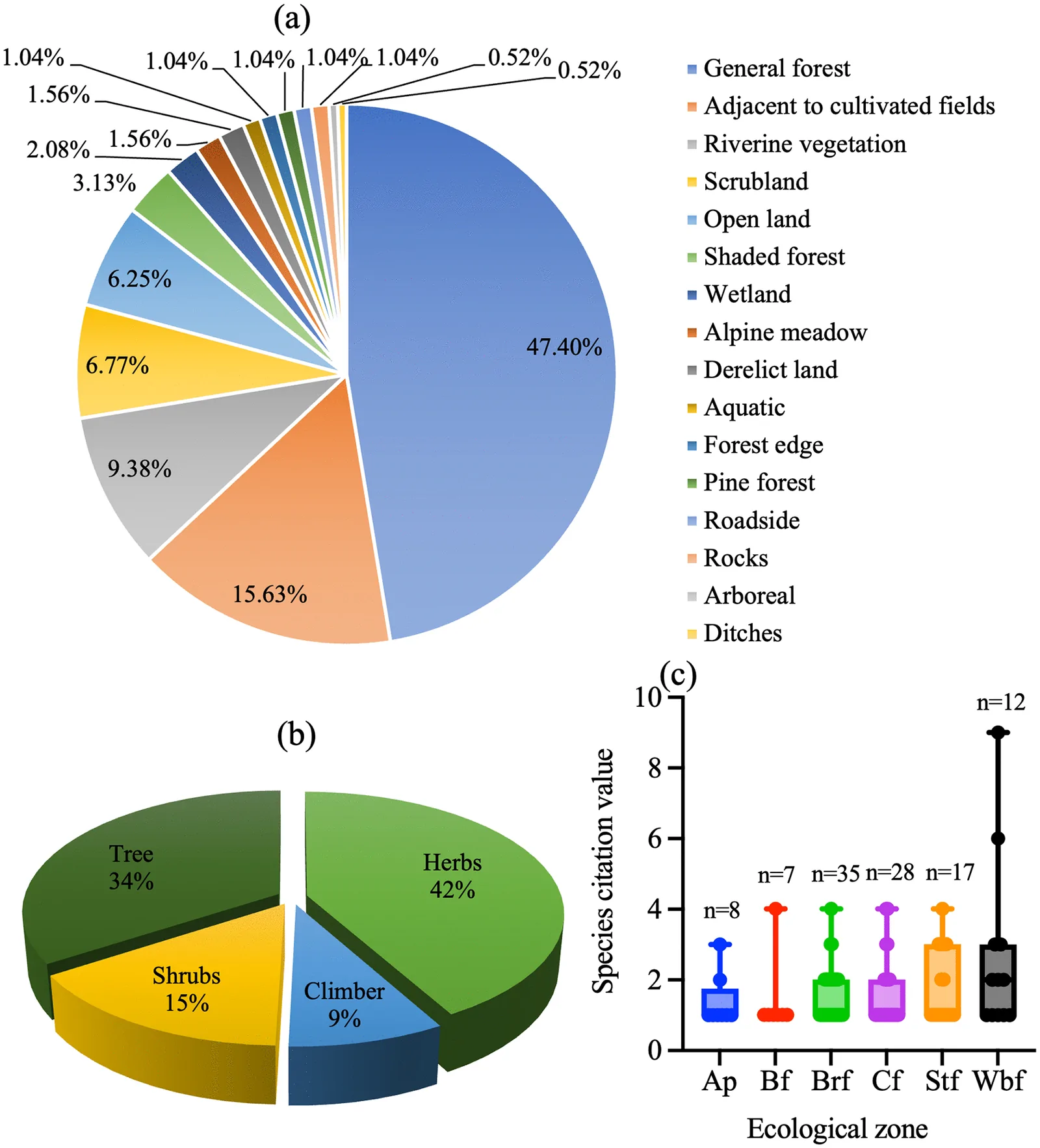
Habitat distribution, life forms, and ecological-zone variation in wild edible plants recorded in Bhutan. (a) Percentage distribution of habitats from which wild edible plants were collected, showing the predominance of general forest habitats, followed by sites adjacent to cultivated fields and riverine vegetation. (b) Life forms among recorded wild edible plants are dominated by herbs, followed by trees, shrubs, and climbers. (c) Distribution of species citation values across the six sufficiently represented ecological zones included in the inferential comparison. Boxes show the median and interquartile range, whiskers indicate the range, points represent individual species, and sample sizes are shown above each box. Citation values did not differ significantly among ecological zones (Kruskal-Wallis χ^2^ = 5.12, df = 5, p = 0.402). Abbreviations: Ap, alpine; Bf, blue pine forest; Brf, broad leaf forest; Cf, chir pine forest; Stf, subtropical forest; Wbf, warm broad leaf forest.

Beyond taxonomic importance, the habitat distribution of wild edible plants also reflects their ecological and functional diversity. These species occur across a wide range of environments, from lowland to highland regions, including forests, fallow lands, riverine vegetation, scrublands, open lands, wetlands, alpine meadows, and disturbed habitats. Notably, a substantial proportion (47.5%) of WEPs were collected from forest ecosystems, followed by abandoned fields and riverine vegetation (Fig 2a). In terms of life forms, herbs dominated (42%), followed by trees (34%), shrubs (15%), and climbers (9%) (Fig 2b), indicating a strong reliance on both understory and woody vegetation components (Table 1).

This ecological dependence has important implications for conservation. The findings of the present study indicate that WEP utilization is closely linked to habitat availability and is increasingly constrained by locally perceived environmental pressures. A large proportion of WEPs are harvested from forest habitats that are vulnerable to disturbance and degradation. In addition, respondents identified ecological change, access limitations, and disturbances (including fire and human or animal interference) as key factors influencing WEP availability (Table 5). These constraints are further reflected in utilization trends, as a notable proportion of respondents reported declining WEP use over time (Fig 5).

Collectively, these patterns indicate that WEP conservation in Bhutan is not merely a general or theoretical concern but a locally experienced issue shaped by habitat dependence, environmental change, and shifting use patterns [37–39]. Preserving and revitalizing WEPs therefore requires integrated strategies that combine conservation, sustainable harvesting, and protection of traditional ecological knowledge. Approaches such as agroforestry, seed banking, and community-based conservation programs offer practical pathways to strengthen the long-term resilience and sustainability of these resources [40,41]. In addition, integrating WEPs into contemporary food systems through education and sustainable commercialization may reinforce their cultural value while supporting biodiversity conservation.

### Use patterns of WEPs

**Fig 3:**
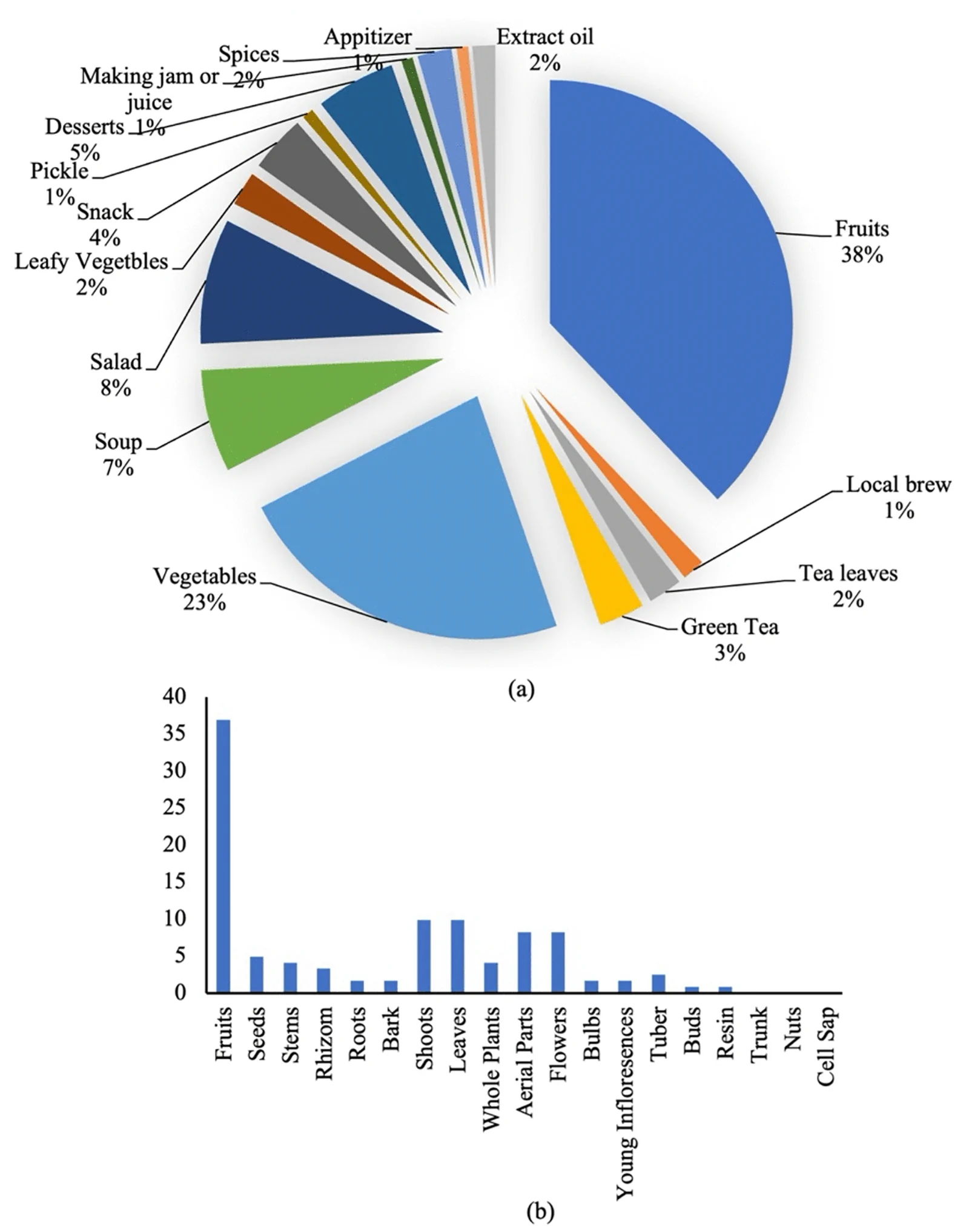
Percentage distribution of WEP consumption modes (a) and plant parts used as food (b).

**Fig 4:**
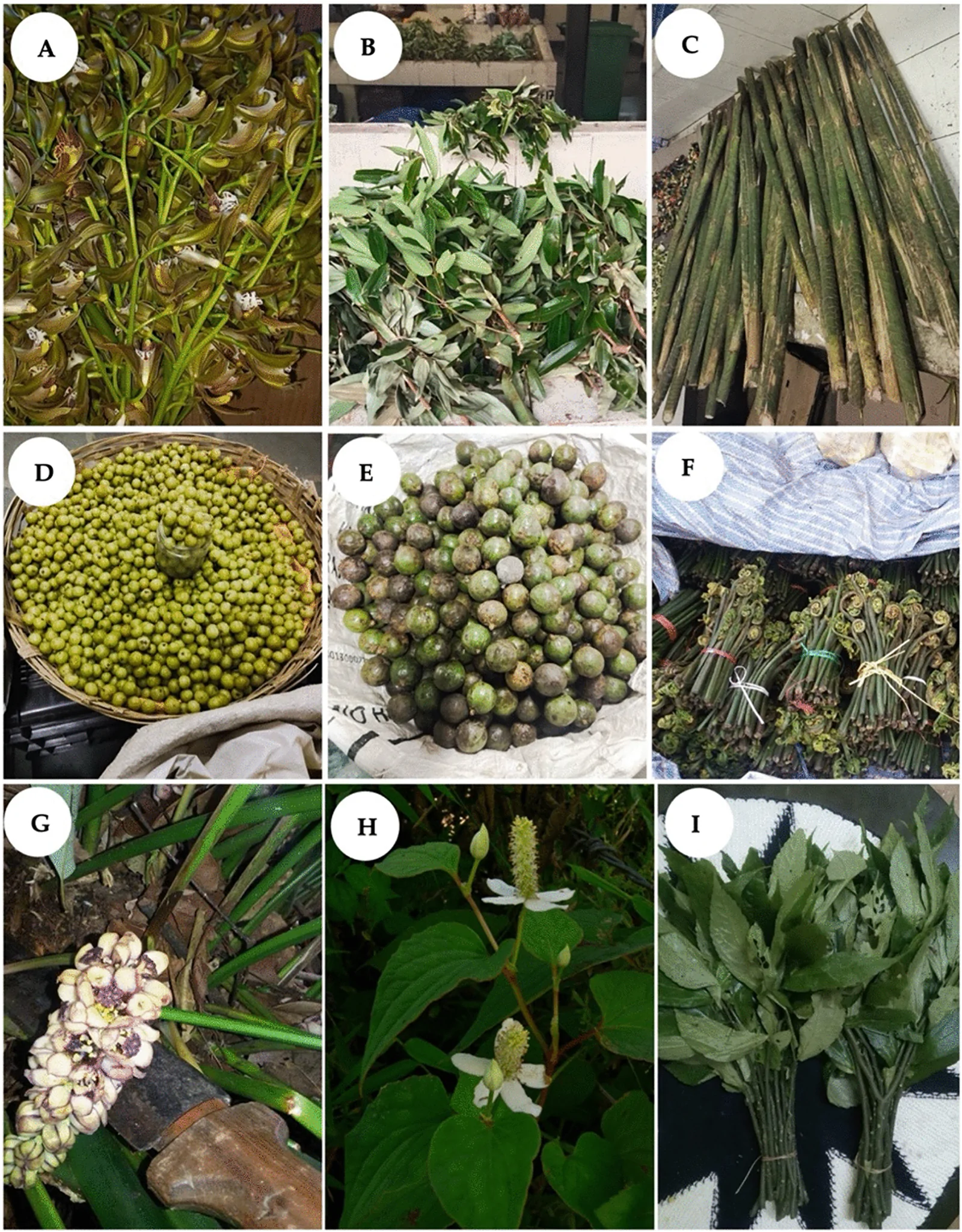
Selected wild edible plants commonly sold in local markets: (A) *Cymbidium floribundum*; (B) *Cinnamomum zeylanicum*; (C) *Plectocomia himalayana*; (D) *Phyllanthus emblica*; (E) *Machilus edulis*; (F) *Diplazium maximum*; (G) *Tupistra nutans*; (H) *Houttuynia cordata*; (I) *Elatostema lineolatum.*

Recorded WEPs spanned multiple food categories, but use was concentrated in fruits and vegetables rather than evenly distributed across all categories (Fig 3a; Table 1). Fruits accounted for the largest share of reported consumption modes (38%), followed by vegetables (23%), and fruit was also the single most frequently used plant part (36.89%) (Fig 3b). This pattern indicates that current WEP use in Bhutan is centered on a relatively small set of nutritionally and culturally important food types. The prominence of widely traded taxa further shows that WEPs contribute simultaneously to household consumption, local exchange, and culinary identity in Bhutan [42]. Examples include Cymbidium floribundum, Cinnamomum zeylanicum, Plectocomia himalayana, Phyllanthus emblica, Machilus edulis, Diplazium maximum, Houttuynia cordata, and Elatostema lineolatum [43]. Rather than functioning as marginal foods, these taxa form part of an active food system that links nutrition, markets, and local food practices [44,45]. Comparable evidence from broader reviews and market studies shows that WEPs often combine dietary, commercial, and cultural functions rather than serving only as fallback foods [4,46]. These representative market taxa are shown in Fig 4.

### Trends in the usage of wild edible plants

Globally, forest resources and their products remain vital to the livelihoods of a large share of the world’s population [47]. In Bhutan, people from diverse communities collect wild edible plants for both nutritional and medicinal purposes. Several respondents expressed concern about declining abundance and availability of these plants in adjacent forests, noting that some now travel for more than an hour to collect them. More broadly, WEP consumption often increases under conditions of food scarcity, poverty, and extreme climatic events [48]. In many developing countries, limited access to adequate food supplies continues to drive reliance on wild edible plants [49].

Wild edible plants therefore remain important to food security in rural communities worldwide. Incorporating them into diets also aligns with the United Nations Sustainable Development Goal of ending hunger [50]. WEPs are recognized sources of vitamins, minerals, and other essential nutrients, providing sustenance and micronutrients to rural households throughout the year [5,16,17]. Numerous studies have demonstrated their importance for food and nutrition security [51,52]. However, the specific nutritional profiles of many reported WEPs remain unknown [53].

One study reported the mineral composition of five WEPs, with fiber content ranging from 2.7 to 12.5 g/100 g and protein content ranging from 1.8 to 6.8 g/100 g. Similarly, research showed that the rhizomes of *Dioscorea* species contain five times more protein and fiber than potatoes and sweet potatoes [54]. Despite these findings, the actual contribution of WEPs to daily dietary requirements remains insufficiently understood and warrants further investigation [28].

**Fig 5:**
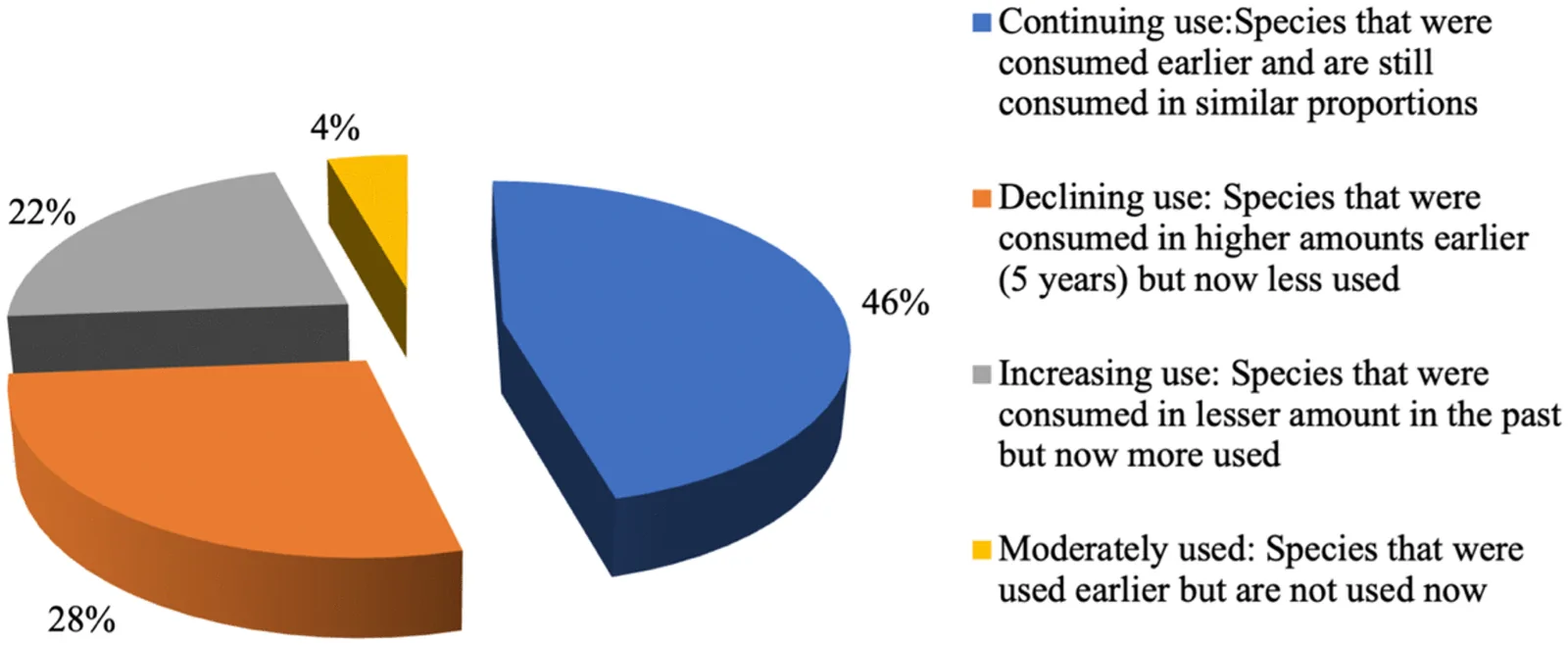
Overall utilization trends of wild edible plants in Bhutan.

To this end, collecting wild edible plants has long been part of Bhutanese farming livelihoods [44]. This practice functions as a social safety net under variable agricultural conditions, as rural households draw on diverse WEPs to offset shortfalls in farm production [52]. Consequently, these plants contribute not only to subsistence but also to household income. Overall utilization trends are shown in Fig 5 and summarized in Table 4, where continuing use was the most frequently reported trajectory (46%).

**Table 4:** Utilization trends across WEP food categories (column totals: increasing n = 56; continuing n = 95; declining n = 45; moderate n = 10).

WEP food category	Increasing use		Continuing use		Declining use		Moderate use	
	n	%	n	%	n	%	n	%
Desserts	4	7.14	0	0	2	4.44	0	0
Extract oil	0	0	0	0	3	6.67	0	0
Fruits	14	25	34	35.79	23	51.11	6	60
Green tea	1	1.79	5	5.26	1	2.22	0	0
Jam or juice	1	1.79	2	2.11	1	2.22	1	10
Pickle	0	0	1	1.05	0	0	0	0
Salad	5	8.93	4	4.21	4	8.89	0	0
Snack	2	3.57	4	4.21	0	0	0	0
Soup	5	8.93	4	4.21	2	4.44	0	0
Spices	1	1.79	3	3.16	0	0	0	0
Tea leaves	3	5.36	0	0	1	2.22	0	0
Vegetables	20	35.71	38	40	8	17.78	3	30

Table 4 summarizes 206 categorical responses across food categories, comprising 56 increasing, 95 continuing, 45 declining, and 10 moderate responses. Use trajectories were mixed, with continuing use the most frequently reported pattern (46%), followed by declining use (28%), increasing use (22%), and moderate use (4%) (Fig 5). Because these data are categorical frequencies, a chi-square test of independence provided an appropriate inferential assessment of whether utilization trends varied across food categories. The test showed a significant association between WEP category and utilization trend (chi-square = 47.84, df = 33, p = 0.046), indicating that increasing, continuing, declining, and moderate use were not evenly distributed across categories. Respondents linked declining use to domesticated alternatives, environmental change, shifting food preferences, and reduced knowledge of species identification, particularly among younger generations [46,55]. At the same time, WEPs continue to function as a support resource for rural livelihoods in Bhutan [44].

**Fig 6:**
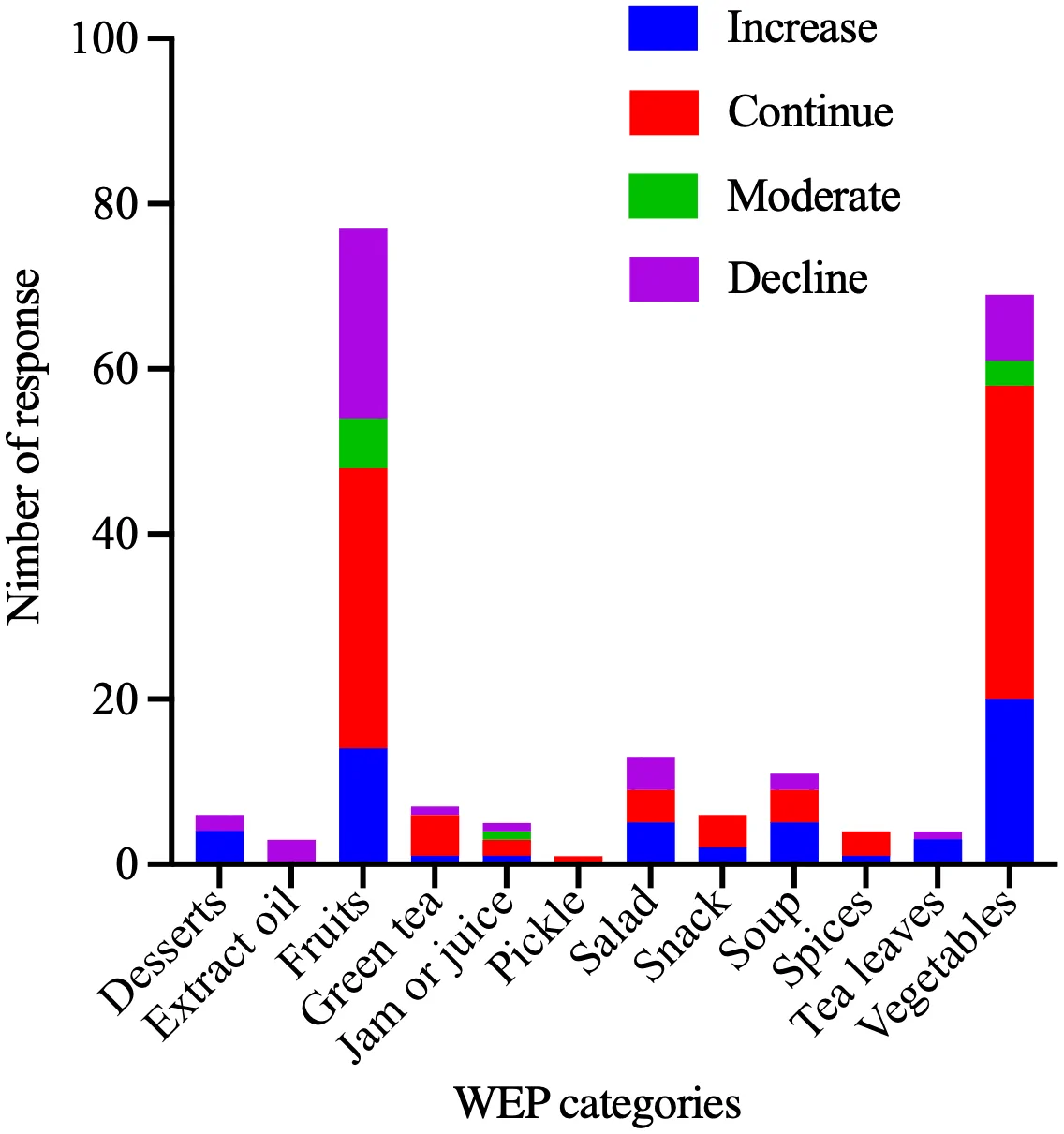
Distribution of utilization trends across WEP food categories in Bhutan. Stacked bar charts show the frequency of responses (n) for four utilization trends: increasing, continuing, moderate, and declining use. Each bar represents a specific food category, with segment heights indicating the number of informant responses for each trend. The results highlight category-specific patterns in the persistence and decline of WEP use.

Column totals in Table 4 show that fruits and vegetables together accounted for 60.71% of increasing-use responses (34/56), 75.79% of continuing-use responses (72/95), 68.89% of declining-use responses (31/45), and 90.0% of moderate-use responses (9/10). Vegetables dominated the increasing and continuing classes, whereas fruits dominated the declining class. These patterns indicate that the food categories most central to everyday WEP consumption are also those undergoing the clearest change. This interpretation is consistent with studies that similarly identify fruits and vegetables as dominant WEP categories and as especially sensitive to ecological and social change [56]; see also (58, 59) and [57,58]. Comparable concentration of use around fruits, leaves, and vegetable species has been reported in broader WEP syntheses and regional food-system studies from Africa, the Hindu Kush, and southern Europe [59–61]. These category-specific differences are illustrated in Fig 6.

The significant category-level association therefore adds inferential support to the descriptive pattern: change in WEP use is concentrated in the most widely used food categories rather than dispersed across the entire repertoire. This suggests a reorganization of use within the core food system rather than a uniform retreat from wild foods. The observed decline in some categories is consistent with broader reports that shifts in access, preferences, and harvesting conditions can affect the persistence of everyday WEP use [62,63]. Similar reorganization of wild food use in response to social and environmental change has been documented in Spain and West Sumatra [64,65].

Taken together, the results suggest that traditional WEP knowledge is not disappearing uniformly but may be transmitted less securely where access declines and regular use is interrupted. In this sense, the reported decline reflects both ecological pressure and weakening opportunities for intergenerational learning. Comparable evidence from Guatemala showed that WEP knowledge was transmitted mainly through relatives, and that people who learned through family networks knew more plants and recognized them better than those learning through other pathways [66]. Comparable generational change linked to reduced forest access has also been documented among Mapuche communities in Chile [67].

### Socio-ecological drivers of WEP use

A total of 922 coded responses were provided by 192 informants regarding factors that promote or constrain WEP use. Because Table 5 summarizes categorical response frequencies rather than continuous measurements, inferential assessment focused on chi-square goodness-of-fit tests within each domain. Domain totals were 187 environmental, 186 sociocultural, 184 agricultural and land-use, 182 economic, and 183 human-wildlife conflict responses (Table 5).

**Table 5:** Socio-ecological motivations and constraints influencing WEP use (coded responses, N = 922).

Motivation category	Characteristics		Frequency (n)	%
Environmental	Explanations related to factors such as climate, abundance, and scarcity	1. Highly available	99	10.74
		2. Difficult to access	21	2.28
		3. Moderately available	14	1.52
		4. Rare/scarce	10	1.08
		5. Environment-driven scarcity	39	4.23
		6. Human-induced scarcity	4	0.43
Sociocultural	Explanations related to taste, aroma, flavor, health, ritual, and interest	7. Difficult to collect	3	0.33
		8. Lack of knowledge	14	1.52
		9. Local food	75	8.13
		10. Medicinal value	40	4.34
		11. Traditional practices	54	5.86
Agricultural and land-use practices	Explanations related to changing agriculture and land use, such as the cultivation of cash crops, etc.	12. Nutritional supplement	10	1.08
		13. Land use change	82	8.89
		14. Religious/nutritional demand	18	1.95
		15. Cultural dietary influence	8	0.87
		16. Medicinal value	24	2.60
		17. No side effects	14	1.52
		18. Other factors	28	3.04
Economic	Explanations about factors such as price, market value, and commercial availability	19. Free to collect	146	15.84
		20. Primary income	12	1.30
		21. Secondary income	12	1.30
		22. Tertiary income	12	1.30
Human-wildlife conflict	Explanations related to crop depredation by wild animals, attacks by wild animals on humans, etc.	23. Animal destruction	43	4.66
		24. Animal and human disturbance	11	1.19
		25. Fire impact	5	0.54
		26. Human disturbance	86	9.33
		27. Neutral impact	19	2.06
		28. No observed damage	19	2.06

**Fig 7:**
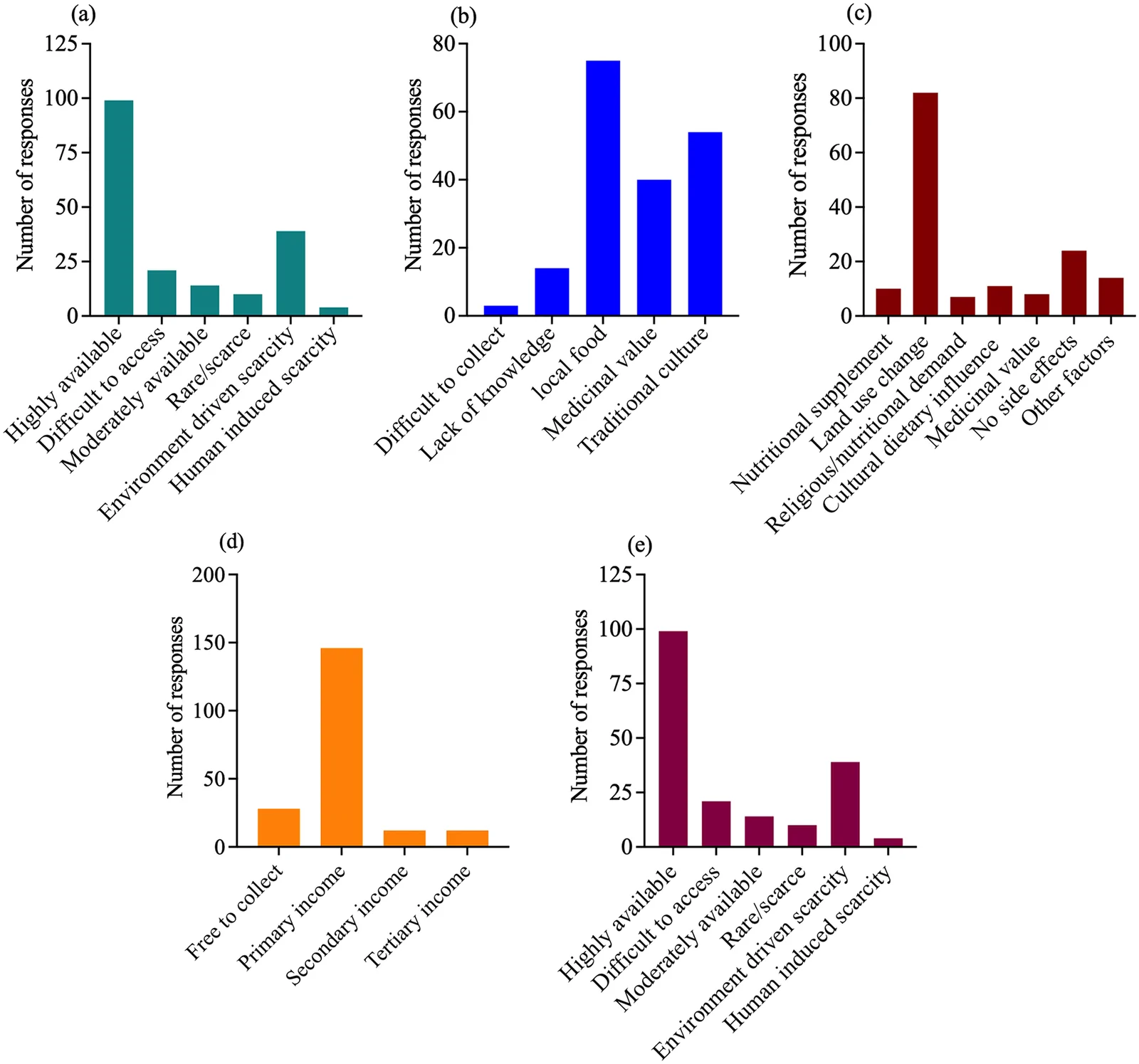
Distribution of key socio-ecological drivers influencing wild edible plant (WEP) utilization in Bhutan. Bar charts represent the frequency of informant responses (n) across five domains: (a) environmental factors, (b) sociocultural drivers, (c) agricultural and land-use practices, (d) economic factors, and (e) human-wildlife conflict. Within each panel, bars correspond to specific explanations, with heights indicating the number of responses associated with each factor. Across domains, responses were unevenly distributed, with a limited number of dominant drivers accounting for most reported influences on WEP utilization.

Responses within every domain were strongly uneven rather than uniformly distributed: environmental (chi-square = 200.43, df = 5, p < 0.001), sociocultural (chi-square = 92.12, df = 4, p < 0.001), agricultural and land-use (chi-square = 149.57, df = 6, p < 0.001), economic (chi-square = 295.98, df = 3, p < 0.001), and human-wildlife conflict (chi-square = 148.57, df = 5, p < 0.001). High availability dominated the environmental domain, local food preference, traditional practices, and medicinal value dominated the sociocultural domain, land-use change dominated the agricultural and land-use domain, free collection dominated the economic domain, and human disturbance dominated the human-wildlife conflict domain. These results show that WEP utilization in Bhutan is shaped by a limited number of dominant motivations and constraints rather than evenly distributed influences. These domain-level patterns are illustrated in Fig 7.

Table 5 strengthens the interpretation of Table 4 by showing that change in WEP use is tied to a concentrated set of socio-ecological drivers. The prominence of local food preference, traditional practices, and medicinal value indicates that continued use in Bhutan is sustained not only by subsistence need but also by taste, cultural embeddedness, and the food-medicine interface. Similar patterns have been reported elsewhere, where the cultural significance of wild foods and the persistence of taste-based gathering practices help maintain use despite broader socioeconomic change [62,63]; see also [68]. The importance of taste, culinary memory, and food-medicine links in maintaining WEP use has likewise been emphasized in Italy and other Mediterranean settings [2,69].

At the same time, the strong signals for land-use change, environment-driven scarcity, and disturbance indicate that declining use is being shaped by both ecological pressure and changing livelihood conditions. This interpretation is consistent with studies showing that wild food use becomes less secure when access changes, habitats are modified, or harvesting conditions deteriorate [62,70]; see also [57,58]. In the present study, this is reflected most clearly in fruits and vegetables, which remained the dominant food categories but also accounted for most declining-use responses.

The dominance of free collection in the economic domain suggests that WEPs continue to provide a low-cost supplement to household diets and small-scale livelihoods, while the prominence of local food and traditional practices shows that their value is simultaneously economic and cultural. Comparative studies likewise note that wild foods can support both subsistence buffering and livelihood resilience, especially where cash resources are limited [71,72]. Overall, the statistical pattern supports interpreting WEP change in Bhutan as a reorganization within the most important food categories, driven by interacting ecological, sociocultural, and economic pressures rather than by simple abandonment of wild foods [73]; see also [74].

### Preservation and governance of WEPs

Wild edible plants (WEPs) are recognized as an economical and nutritious food source that contributes substantially to the global food supply, particularly in Himalayan regions [1,75]. In Bhutan, rural communities use WEPs for household consumption and income generation through local marketing [16,76,77]. Their value also derives from their nutritional content, including proteins, vitamin B2, and vitamin C, which makes them viable supplements to plant-based diets [78,79]. Despite their role in food security, WEPs are often neglected and stigmatized as foods for poorer households [5]. However, several studies have noted declining accessibility of WEPs, because of habitat degradation, land-use and forest-management change, overexploitation, changing dietary preferences, rapid development, and erosion of traditional ecological knowledge [80–83], and similar trends have been reported in Bhutan [19,44].

Growing interest in local products and the commercialization of high-value or rare WEP species by farmers has led to unsustainable extraction and declining populations [71,72]. For example, unsustainable harvesting has pushed popular species such as *Gnetum buchholzianum* and *G. africanum* toward vulnerable status. Likewise, species of *Dioscorea* have shown declining populations in Nangkor Gewog, Zhemgang [84], while *Pogostemon amaranthoides* Benth. and *Persea bootanica* (Meisn.) Kosterm. have declined in Maenbi Gewog, Lhuntse, because of overexploitation and destructive collection practices such as branch cutting [45]. These cases highlight the need to control overexploitation, promote environmentally responsible harvesting methods, and prevent illegal collection of rare WEP species. The herb gardens established in Lingshi (4,000 masl) and Lingmithang (600 masl) for ex situ conservation of crops, medicinal plants, and aromatic plants demonstrate the value of similar approaches for important and rare WEPs [85]. Likewise, in situ conservation in the Yakpugang Community Forest in Kelikhar, Mongar District, eastern Bhutan, illustrates the value of diversifying these approaches nationwide (86). Long-term sustainability will therefore depend on coordinated off-site and on-site conservation, domestication, and management strategies involving government agencies, local communities, and individual resource users [16,19,71,84].

Numerous studies show that the preservation and utilization of WEPs extend beyond botanical or ecological concerns and play an important role in supporting the quality of life of rural communities in many developing regions. WEPs are particularly important during periods of food shortage and continue to form part of everyday diets for millions of people worldwide, providing essential minerals and nutrients [86]. Given their significance, further research is needed to update inventories and document their availability and use among Bhutanese communities. Involving local communities in conservation and management is also crucial because they are the primary custodians and users of these resources. WEP fairs and food festivals may further strengthen awareness, renew interest among younger generations, and encourage active community participation in resource management. In addition, cultivation and domestication through appropriate modern approaches will be important for maintaining future availability.

## Conclusion

This national assessment recorded 114 WEP species in 61 families and shows that WEP use in Bhutan remains important but unevenly distributed. Herbs were the dominant life form, fruits were the most frequently used plant part, and fruits and vegetables remained the core food categories in current use. The results suggest that WEP use is being reconfigured rather than uniformly abandoned: continued use is supported by accessibility, local food traditions, and perceived medicinal value, whereas land-use change, perceived scarcity, and limited species knowledge constrain use. Pressure on both availability and knowledge transmission appears greatest where WEP use depends on forest habitats and younger generations are less engaged in plant identification and use. To support the long-term sustainability of WEPs, the study recommends:

i Integrating WEP knowledge into school curricula and community education programs to engage younger generations;
ii Supporting community-based conservation and sustainable harvesting initiatives;
iii Promoting value-added processing and marketing of WEPs to enhance livelihoods; and
iv Strengthening policy support for preserving indigenous knowledge systems.

Together, these actions can help sustain WEP use, preserve cultural identity, strengthen food and nutrition security, and provide a clearer foundation for future research and conservation planning.

## Abbreviations

WEPs: Wild edible plants;
Masl: Meters above sea level
FAO: Food and Agriculture Organization
UV: Use Value
ICF: Informant Consensus Factor
RFC: Relative Frequency Citation
FIV: Family Importance Value
FL: Fidelity Level
